# Person-centered strategies for integrating TB treatment into community pharmacies for people with TB/HIV in Uganda: A human-centered design methodology study protocol

**DOI:** 10.1371/journal.pone.0344913

**Published:** 2026-03-12

**Authors:** Jonathan Izudi, Adithya Cattamanchi, Christine Sekaggya-Wiltshire, Rachel King, Noah Kiwanuka, Amanda Sammann

**Affiliations:** 1 Directorate of Graduate Training, Research and Innovation, Muni University, Arua, Uganda; 2 Makerere University Infectious Diseases Institute, Kampala, Uganda; 3 Department of Community Health, Faculty of Medicine, Mbarara University of Science and Technology, Mbarara, Uganda; 4 Division of Pulmonary Diseases and Critical Care Medicine, University of California Irvine, Irvine, California, United States of America; 5 Center for Tuberculosis, Institute for Global Health Sciences, University of California San Francisco, San Francisco, California, United States of America; 6 Mulago National Referral and Specialized Hospital, Kampala, Uganda; 7 School of Medicine, St. Andrews University, Scotland, United Kingdom; 8 Institute for Global Health Sciences, University of California, San Francisco (UCSF), San Francisco, California, United States of America; 9 Department of Epidemiology and Biostatistics, School of Public Health, Makerere University, Kampala, Uganda; 10 Department of Surgery, University of California, San Francisco (UCSF), San Francisco, California, United States of America; University of Ghana College of Health Sciences, GHANA

## Abstract

**Background:**

Community pharmacies (private retail drug shops or pharmacies) have successfully delivered antiretroviral therapy (ART) to people with human immunodeficiency virus (HIV) and could support integrated tuberculosis (TB) treatment, but the implementation strategies are unclear. To inform a planned pilot randomized trial, we aim to develop person-centered strategies for integrating TB treatment into community pharmacies targeting people with TB/HIV using a Human-Centered Design (HCD) methodology. Here, we describe the study protocol.

**Methods:**

We will employ a three-phased HCD methodology comprising inspiration, ideation, and implementation across six primary health facilities in Kampala, Uganda. Eligible participants will include people with TB/HIV, focal persons for TB and HIV, Ministry of Health officials, and community pharmacy healthcare providers. The inspiration phase will build the themes on barriers and facilitators to integrating TB treatment into community pharmacies from a qualitative study, complemented by participant observations at selected 2–3 community pharmacies to understand the care pathway of people with TB/HIV (journey mapping), including sketching the ideal pharmacy-based TB treatment pick-up. The ideation phase will use design workshops to consolidate identified themes, generate insight statements, including translating them into design opportunities, and conclude with forming low and high-fidelity prototypes. The implementation phase will comprise two rounds of prototype testing, low and high fidelity, with 12–16 participants per round, including people with TB/HIV, focal persons, Ministry of Health officials, and pharmacy health workers. Feedback on usability, desirability, feasibility, and viability will guide iterative refinement, with high-scoring prototypes in system usability surveys prioritized for trial.

**Discussion:**

Through iterative user engagement, we will adapt contextually relevant strategies that will leverage key facilitators and address barriers to TB treatment integration. Strategies demonstrating perceived usefulness, user-friendliness, high acceptability, person-centeredness, and contextual relevance will be adapted and piloted in a planned randomized trial aiming to determine feasibility, acceptability, and fidelity, including preliminary effectiveness.

## Introduction

Tuberculosis (TB) and human immunodeficiency virus (HIV) are the leading causes of morbidity and mortality globally [1]. Optimal treatment outcomes among people with TB/HIV contribute to reducing TB morbidity and mortality, including transmission in the community and ending the TB epidemic [2]. The estimated global treatment success rate among people with TB in 2023 is 88% and around 79% among those with HIV [3]. In sub-Saharan Africa, the rate is 70.1% among people with TB/HIV based on data from a systematic review and meta-analysis [4]. In Uganda, treatment success rates among people with TB/HIV across studies in Uganda range between 54.5% and 74% [5–8]. The World Health Organization (WHO) desired threshold for treatment success rate is ≥ 90%. Therefore, there is a need for innovative approaches to optimize the treatment rates.

In high TB burden settings such as Uganda, missed clinic visits and extended travel distances remain major hindrances to achieving optimal treatment success and adherence among people with TB/HIV. Evidence from a previous Ugandan study showed that people with TB/HIV who travel ≥5 km to a TB clinic have a 17% lower likelihood of achieving treatment success compared to those who travel <5 km [9]. Another study in central Uganda indicated that the mortality rate among people with TB and/or HIV who travel ≥2 km to a TB clinic ranges from 9% to 27% higher when compared with those who travel <2km to the same clinic [10]. Community pharmacies (private retail drug shops or pharmacies) have emerged as innovative and promising platforms for refilling antiretroviral therapy (ART) among people with HIV in Uganda and have registered remarkable viral load suppression and treatment adherence rates. Studies have reported ≥95% ART adherence [11] and ≥95% viral load suppression rates [12] among people with HIV who receive ART refills through community pharmacies. These findings demonstrate community pharmacies as a successful differentiated service delivery model for ART delivery and underscore the potential for integrating TB treatment for people with TB/HIV.

Although community pharmacies show promise for delivering person-centered TB care, several uncertainties remain around the optimal timing of integration, implementation strategies, and real-world effectiveness.

To address these uncertainties and inform the implementation of a planned randomized trial, we designed the Community Pharmacy Tuberculosis Treatment (COPHAT) study, which proposes to use Human-Centered Design (HCD) methodology to adapt person-centered strategies for integrating TB treatment into community pharmacies targeting people with TB/HIV in Kampala, Uganda. Here, we describe the HCD methodology.

HCD offers an innovative, person-centered approach to developing contextually relevant implementation strategies [13]. Through interactive and participatory decision-making among stakeholders [14], HCD aims to improve the acceptability, appropriateness, and feasibility of proposed interventions. Although HCD has been applied to enhance the implementation of interventions for HIV and TB care [15–18], most studies have not published their protocols, which hinders replication and adaptation in other settings. We address this gap by providing a detailed account of our planned application of HCD to TB/HIV intervention design to advance methodological transparency and support the wider adoption of HCD to strengthen person-centered TB/HIV service delivery in high-burden settings.

## Methods and materials

### Study design and setting

We propose to use HCD methodology, a novel, person-centered approach to design thinking, to develop context-relevant implementation strategies [13] and support integration of TB treatment into community pharmacies. HCD methodology supports the generation of meaningful, feasible, and effective solutions to address problems prioritized by stakeholders [13]. The methodology entails an interactive process to achieve shared decision-making by incorporating feedback from stakeholders [14], allowing rapid and iterative co-design and testing of context-relevant strategies before the implementation.

This study will be conducted at all six purposively selected primary healthcare facilities in Kampala, the Capital city of Uganda. The rationale for selecting the facilities is that their HIV clinics are affiliated with some community pharmacies to provide ART refills to stable people with HIV, as part of the Uganda Ministry of Health differentiated service delivery model. Each health facility has a focal person for TB and HIV—a healthcare provider responsible for TB and HIV services delivery, respectively.

The TB clinic is headed by the TB focal person, while the HIV clinic is headed by the HIV focal person. Both focal persons have substantial work experience, training, and knowledge in their respective fields. The clinics provide standardized care per the national treatment guidelines and operate five days a week, with access available during weekends for individuals needing urgent care. The health facilities identify eligible people with HIV who require pharmacy ART refills, assist them in selecting a community pharmacy of their choice, share their unique identifiers with the selected pharmacies, and periodically supply the pharmacies with ARVs for dispensing. In contrast, people with TB/HIV receive anti-TB refills through the TB clinic until treatment completion. These health facilities are described in our previous studies [19–22].

### Study participants and sampling

We will interview all relevant participants (stakeholders), including people with TB/HIV and healthcare providers—TB and HIV focal persons, MoH technical experts, and community pharmacy healthcare providers. The enlisted healthcare providers are engaged in the integration of ART into community pharmacies, so they have a good understanding and experience of the integration processes. The eligible people with TB/HIV will be those aged ≥18 years, have been on both TB and HIV treatment for ≥2 months, and are receiving ART refills either through the community pharmacies or health facilities. The two-month threshold ensures participants have transitioned beyond the initial intensive phase of TB treatment and have adequate exposure to their respective medication refill models, allowing them to provide meaningful insights. We will consecutively sample them across the six TB clinics. During the data collection period, trained research assistants will screen all individuals attending TB/HIV clinic appointments for eligibility, and eligible persons will be provided with study information and invited to participate until the required sample size at each facility is attained. Our sampling approach ensures that all eligible individuals at different stages of their treatment who present to the clinics during the study period are included following informed consent. People with TB/HIV who are acutely ill, require urgent medical care, or are otherwise unable to participate in the interview will be excluded.

Eligible healthcare providers will be those with ≥6 months of work experience in TB management. We will purposively sample TB and HIV focal persons and MoH officials as key informants. For each health facility, we will randomly select one affiliated community pharmacy serving approximately ≥50 people with HIV, and at the selected pharmacy, we will purposively sample 1–2 pharmacy healthcare providers for interviewing.

The pharmacy healthcare providers will be those who have worked in the pharmacy for ≥2 months and are engaged in pharmacy-based ART refills to ensure they have adequate experience and are familiar with the refill process. TB focal persons on leave during the study period and Ministry of Health officials unavailable during data collection will be excluded. Pharmacy personnel not routinely involved in ART refill services, including those temporarily covering shifts, will be excluded.

### Procedures

**Fig 1:**
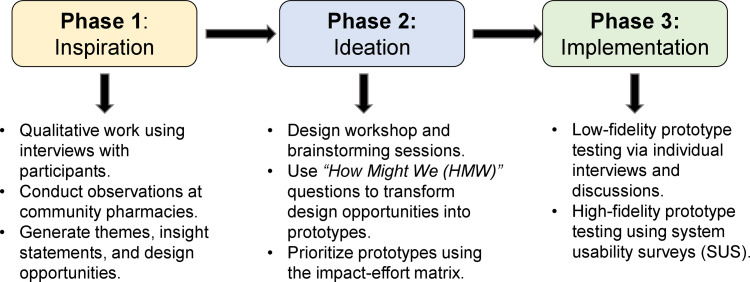
Human-Centered Design (HCD) methodology phases for adapting implementation strategies to integrate TB treatment into community pharmacies for people with TB/HIV in Kampala, Uganda. The diagram illustrates the three sequential stages of the methodology, along with the corresponding main activities.

As shown in Fig 1, the HCD methodology will follow three distinct phases: inspiration, ideation, and implementation [23]. This process is conceptually grounded in the Double Diamond model of design, which emphasizes iterative cycles of divergence and convergence to structure problem exploration and solution development [24]. Fig 1. Human-Centered Design (HCD) methodology phases for adapting implementation strategies to integrate TB treatment into community pharmacies for people with TB/HIV in Kampala, Uganda.

The inspiration phase aligns with the “Discover” and “Define” stages of the Double Diamond, where insights from qualitative data are explored broadly (divergence) and then synthesized into priority themes and insight statements (convergence). The ideation phase corresponds to the “Develop” stage, characterized by generating and refining potential solutions through iterative expansion and narrowing of design options. The implementation phase aligns with the “Deliver” stage, focusing on testing, refining, and operationalizing selected strategies within real-world pharmacy settings.

### Phase 1: Inspiration phase (qualitative work—interviews and observations)

The inspiration phase will build on our prior qualitative work that identified barriers and facilitators to integrating TB treatment into community pharmacies [25]. Two reviewers (JI and AS) will independently review and select themes on identified barriers and facilitators to integrating TB treatment into community pharmacies, which will be reviewed and approved by a third senior reviewer (AC). Once consensus has been established regarding the key themes amongst the reviewers (JI, AS, and AC), we will generate insight statements from the themes to capture user perspectives, motivations, and tensions, allowing us to define specific human needs.

The generation of insight statements is specific to HCD methodology and is an academically novel approach to assigning meaning to themes. The three reviewers (JI, AS, and AC) will review, discuss, and refine the insight statements until consensus is reached. Building on the insight statements, we will develop design opportunities—propose actionable solutions to identified challenges and develop low-fidelity prototypes for refinement during the subsequent phases of the HCD methodology. The low-fidelity prototypes will also be reviewed, discussed, and refined until consensus is reached. Besides the existing qualitative work, we will conduct observations across 2–3 community pharmacies to understand the care pathway of people with TB/HIV (journey mapping) and sketch the ideal pharmacy-based TB treatment pick-up. The observations will be conducted using a checklist and will focus on five areas: 1) activities: tasks performed, duration, and sequence; 2) the environment: physical characteristics of the pharmacy, functioning and use; 3) interactions: person-to-person and person-to-object engagements; 4) objects: types of objects and their characteristics, including the relation to people and activities, and 5) users: presence, behaviors, roles, and relationships.

### Phase 2: Ideation phase: brainstorming and prototype development

In this phase, we will engage the participants, namely people with TB/HIV, focal persons for HIV and TB, MoH technical experts, and community pharmacy healthcare providers in a design workshop comprising 8–12 people. We will present the identified themes, insight statements, and design opportunities from the inspiration phase using a PowerPoint format to facilitate consolidation. The participants will vote to select 3–4 insights related to challenges they wish to be addressed immediately, and another 3–4 insights for future action. Voting will be conducted privately using individual ballots, and selections will be tabulated by the research team without disclosing individual choices to ensure the process is independent and unbiased. Thereafter, participants will be organized into smaller groups of 3–5 people for brainstorming sessions focused on the selected insights, guided by a *priori* defined 2–3 *“How Might We”* (HMW) questions to generate solutions or prototypes. The brainstorming session will aim to overcome cognitive fixedness—a mindset where one consciously or unconsciously assumes only a one-way approach to a situation. We propose conducting 2–3 brainstorming sessions with 5–10 people with TB/HIV aged ≥18 years, 3–5 focal persons for TB, 3–5 focal persons for HIV, and 3–4 Ministry of Health officials.

Anticipated prototypes may include guideline or standard operating procedure development to support TB treatment integration, multi-month dispensing of TB medications, medication side effect assessment using pre-defined questions, a new pathway for medication pick-ups, and laboratory-only sputum smear follow-up testing, among others. To prioritize and select the prototypes, we will use an impact-effort matrix for plotting ideas based on their perceived impact (low vs. high) and implementation effort (low vs. high). We will select ideas in the high-impact, low-effort quadrant for testing. The identified prototypes will be packaged for iterative testing and refinement in the subsequent phase. To accommodate the participants’ competing responsibilities, the ideation phase may be held off-site at the Makerere University Infectious Diseases Institute boardroom, including weekends. Where feasible, it will be conducted at the health facilities.

### Phase 3: Implementation phase: prototype testing, iteration, and refinement

We will conduct two rounds of prototype testing, low and high fidelity, each involving 12–16 participants deemed sufficient to attain thematic saturation, including people with TB/HIV, TB and HIV focal persons, MoH officials, and pharmacy health workers. In the low-fidelity phase, participants will engage in 60-minute individual interviews with a talk-aloud session to review the prototype, discuss what works, and provide feedback on desirability, feasibility, and viability using a 5-point Likert scale, ranging from a low to high score (1 = Very low, 2 = Low, 3 = Moderate, 4 = High, and 5 = Very high). The participants will also compare 2–3 prototype options and vote for their preferred version. Findings from this phase will guide revisions before the high-fidelity phase, during which usability will additionally be assessed using the System Usability Scale (SUS) using a simple, 10-item standardized questionnaire [26]. Each SUS question will be ranked on a 1–5 scale, with the lowest score indicating a strong disagreement with the prototype and the highest score suggesting otherwise. We will implement the SUS until a higher level of acceptability, defined as a median score of >80.3, is reached [26]. A maximum of three iterative refinement cycles will be conducted to allow systematic improvement of prototypes based on user feedback. Prototypes achieving higher SUS and Likert-scale scores will be prioritized for pilot implementation in a hybrid effectiveness–implementation randomized trial at two public primary healthcare facilities in Kampala, Uganda.

If the predefined SUS threshold of >80.3 is not achieved after the maximum number of iterations, the prototype with the highest median SUS score (provided it meets or exceeds the acceptable usability benchmark of ≥68) and demonstrates consistent improvement across rounds will be selected. Qualitative user feedback and feasibility considerations will additionally inform the final selection to ensure contextual appropriateness. This iterative, user-centered approach ensures systematic refinement and selection of the most acceptable, feasible, and effective intervention prototypes.

### Study timelines

Participant recruitment and data collection will occur from 1 February to 30 May 2026. Data cleaning, verification, and preliminary analysis are planned for June 2026, followed by final statistical analysis and synthesis of results in July 2026. Final results will therefore be available by 31 July 2026.

### Data management

Audio recordings and transcripts will be de-identified using participant codes, and direct identifiers such as names will not be used. Digital files will be stored on password-protected, encrypted computers, and physical documents like consent forms and field notes will be kept in locked cabinets with restricted access. Only the research team will have access to the data. Data used in reporting will be presented in aggregate or with pseudonyms to prevent participant identification. Audio recordings will be securely destroyed after transcription verification, and all data will be retained and disposed of per institutional ethical guidelines. Participants’ time will be compensated in accordance with local ethics regulations, in the form of snacks and refreshments.

### Statistical methods: Sample size and data analysis

The sample sizes for the different phases of the HCD methodology have been described above. However, the exact sample sizes will depend on thematic saturation, a point at which no new information emerges even when additional data are collected. All interviews will be audio-recorded and professionally transcribed verbatim by experienced qualitative researchers. The transcripts will be checked for accuracy by replaying and listening to the audio recordings, and any inconsistencies will be identified and corrected.

The corrected transcripts will be uploaded into NVivo software for thematic analysis using an inductive approach. To minimize subjective bias, two independent analysts (JI and AS) will conduct the analysis. Each analyst will independently and repeatedly read a few of the transcripts to achieve familiarity with the data (data immersion), identify recurring and significant patterns, and develop codes. Through consensus, the analysts will develop an initial codebook based on the independent codes, which will be used to code the remaining transcripts and remain open to newer codes. We will iteratively refine and group the codes into categories and subsequently into themes, and present them alongside illustrative quotes to support interpretation. Reporting of the qualitative findings will adhere to the Standards for Reporting Qualitative Research (SRQR) guidelines [27].

In SUS data analysis, we will normalize the data by subtracting 1 from each odd-numbered question (questions 1, 3, 5, 7, and 9) and 5 from each even-numbered question (questions 2, 4, 6, 8, and 10). This process will create new values, which will be summed to produce a total score, which will be multiplied by 2.5 to yield scores that will range from 0 to 100, with 68 as the average score [26]. We will categorize the SUS scores as ≤50, 51–68, 68–80.3, and more than 80.3 to suggest unacceptable, poor, good, and excellent strategies/prototypes, respectively [28]. Prototypes with SUS scores ≥68 will be considered usable and may be tested in the planned trial.

### Ethics statement

We obtained administrative clearance from the Directorate of Public and Environmental Health, Kampala Capital City Authority (Ref: DPHE/KCCA/1301/01). Ethical approval was obtained from the Makerere University Infectious Diseases Institute Research Ethics Committee (Ref: IDI-REC-2024–98) and the Uganda National Council for Science and Technology (Ref: HS4397ES). Participants will provide informed consent in writing or thumbprint after receiving comprehensive information about the study process, rationale, purpose, potential risks and benefits, and ethics approvals. We will also explain existing measures to protect the privacy and the confidentiality of participant information, including the right to withdraw from the study and compensation for the time spent in the study. All qualitative data will be de-identified and stored on a password-protected, encrypted laptop, with physical documents kept in locked cabinets. Only the research team will have access. Audio recordings will be destroyed after transcription verification, and data will be retained and disposed of following institutional ethical guidelines.

The study will be conducted in accordance with the principles of the Declaration of Helsinki on ethical conduct of research involving human participants.

### Dissemination plan

Study findings will be shared at multiple levels to ensure uptake and impact. At the health facility level, results will be communicated to health workers and people with TB/HIV through validation meetings and health education talks. At the district and national level, synthesized reports and presentations will be shared with the Directorate of Public and Environmental Health, Kampala Capital City Authority (KCCA), the Ministry of Health, and the National TB and Leprosy Control Program (NTLP) to inform policy and program implementation. At the international level, the broader scientific community will be reached through conference abstract presentations and peer-reviewed manuscript publications.

### Patient and public involvement

Patients and community members were not involved in the development of this protocol. Also, no patient and public involvement activities were conducted.

## Discussion

Our study will use the HCD methodology to adapt person-centered strategies for integrating TB treatment into community pharmacies among people with TB/HIV in Kampala, Uganda. The main goal is to develop and adapt implementation strategies that are stakeholder-designed, contextually grounded, feasible, and acceptable. We will follow the steps of the HCD methodology, drawing on barriers and facilitators identified in a qualitative study that employed the consolidated framework for implementation research (CFIR) tool. The CFIR domains will inform the inspiration phase of the HCD methodology by providing a structured framework to interpret qualitative findings. Specifically, CFIR constructs identified from interviews and observations will be mapped to insights, which will then guide the formulation of *“How Might We”* (HMW) questions. These HMW questions will be translated into design opportunities, which will subsequently be used to develop actionable prototypes. This approach ensures that the HCD process is theoretically grounded in implementation science and that each phase systematically builds on CFIR-informed qualitative data.

The rationale for using community pharmacies for delivering TB treatment is based on their potential to deliver person-centered care and improve health system efficiency. Studies have shown that ART integration into community pharmacies for people with HIV reduces the healthcare provider workload [11] and frees up their time to care for individuals who require critical care [29,30]. At the health system level, the approach has led to decongesting health facilities [30]. Therefore, the integration of TB treatment into community pharmacies has several untapped potential. Community pharmacies offer greater convenience by enabling people with TB/HIV to refill both TB and HIV medications at a single location and time, hence reducing both direct and indirect costs associated with treatment access [31]. This flexibility may reduce the risk of unintended TB/HIV status disclosure [32] and mitigate both self and community stigma associated with TB and HIV [11,30,33–35].

Evidence also shows that in urban settings, people with TB/HIV often have unstable or changing physical addresses [34], and community pharmacies offer a more efficient and adaptable service delivery approach when compared with other community-level differentiated service delivery models. Our proposed integration of TB treatment into community pharmacies thus aims to extend these benefits recorded among people with HIV to those with TB/HIV.

In our planned pilot, individually randomized, hybrid type 2 effectiveness-implementation trial, we will primarily assess the effectiveness of integrating TB treatment into community pharmacies. Effectiveness outcomes will include HIV viral load suppression and TB treatment success at six months. Also, we will evaluate key implementation outcomes, including feasibility, acceptability, and fidelity as secondary outcomes. We aim to generate robust evidence to inform and strengthen the efforts of the national TB program in Uganda and similar settings in advancing person-centered care for people with TB/HIV, including contributing to ending the TB epidemic by 2035.

### Study strengths and limitations

A key strength of the study is the planned use of HCD methodology, which emphasizes empathy and direct engagement with participants at various health systems levels: people with TB/HIV at the community level, TB and HIV focal persons and community pharmacy healthcare providers at the health facility level, and MoH officials at the national level. The use of HCD methodology facilitates the identification of context-specific needs, preferences, and challenges that may be overlooked via conventional or researcher-driven methods.

By grounding implementation strategies in the lived experiences of participants or real-world settings, the methodology may enhance the relevance, feasibility, and acceptability of the intervention and its strategies. Besides, the methodology emphasizes co-design and promotes early and active participant engagement to foster ownership and increase strategy adoption and sustained use. The iterative nature of the methodology, through activities such as prototyping and real-time feedback, supports the development of strategies that are responsive and adaptable to the local contexts. Lastly, the methodology inherently amplifies the voices of marginalized or underrepresented groups, thereby contributing to more equitable implementation strategies.

Despite the strengths of HCD methodology, there are certain limitations. The methodology can be time- and resource-intensive, requiring skilled facilitation and multiple engagement rounds, which may be challenging. To mitigate this limitation, we will use experienced and skilled individuals with expertise and training in the methodology. Additionally, most HCD-generated solutions/prototypes are often context-specific and may limit generalizability. There is also the risk of incomplete representation of participant engagement, although our proposed multi-level participant engagement approach will likely mitigate this limitation. In addition to subjective bias during data analysis, the HCD process may be affected by selection bias, as participants attending workshops may differ from those who do not; social desirability bias, where participants provide responses they perceive as favorable; and facilitation or group influence bias, where dominant voices may shape group decisions. To minimize these biases, we will use purposive and consecutive sampling, conduct confidential and independent voting, and employ trained facilitators to ensure equitable participation and capture diverse perspectives. Finally, the methodology is effective at generating user-centered solutions but may not fully address structural or system-level barriers. To mitigate this limitation, we will combine the HCD methodology with CFIR to ensure both user-centeredness and methodological rigor, while enhancing the potential for scalability and sustainability of resulting implementation strategies.

## Conclusion and recommendation

Our proposed HCD methodology will adapt person-centered implementation strategies that are contextually relevant, practical, feasible, and acceptable to all participants. The strategies will inform the integration of TB treatment into community pharmacies in a future pilot randomized trial targeting people with TB/HIV in Kampala, Uganda.

This methodology may be useful in other contexts where integration of services is becoming recommended as global health resources become increasingly limited.
